# A Metabolomics-Guided Exploration of the Phytochemical Constituents of *Vernonia fastigiata* with the Aid of Pressurized Hot Water Extraction and Liquid Chromatography-Mass Spectrometry

**DOI:** 10.3390/molecules22081200

**Published:** 2017-07-27

**Authors:** Keabetswe Masike, Bradley S. Khoza, Paul A. Steenkamp, Elize Smit, Ian A. Dubery, Ntakadzeni E. Madala

**Affiliations:** 1 Department of Biochemistry, University of Johannesburg, P.O. Box 524, Auckland Park 2006, South Africa; keabetswemasike@yahoo.com (K.M.); BSKHOZA@sun.ac.za (B.S.K.); psteenkamp@csir.co.za (P.A.S.); idubery@uj.ac.za (I.A.D.); 2 Department of Chemistry, University of Johannesburg, P.O. Box 524, Auckland Park 2006, South Africa; esmit@uj.ac.za

**Keywords:** *Vernonia fastigiata*, PHWE, chemometrics, clovamide, cloud plot, deoxyclovamide, flavonoids, PCA, XCMS

## Abstract

*Vernonia fastigiata* is a multi-purpose nutraceutical plant with interesting biological properties. However, very little is known about its phytochemical composition and, thus the need for its phytochemical characterization. In the current study, an environmentally friendly method, pressurized hot water extraction (PHWE), was used to extract metabolites from the leaves of *V. fastigiata* at various temperatures (50 °C, 100 °C, 150 °C and 200 °C). Ultra-high performance liquid chromatography-quadrupole time of flight mass spectrometry (UHPLC-qTOF-MS) analysis in combination with chemometric methods, particularly principal component analysis (PCA) and liquid/gas chromatography mass spectrometry (XCMS) cloud plots, were used to descriptively visualize the data and identify significant metabolites extracted at various temperatures. A total of 25 different metabolites, including hydroxycinnamic acid derivatives, clovamide, deoxy-clovamide and flavonoids, were noted for the first time in this plant. Overall, an increase in extraction temperature resulted in an increase in metabolite extraction during PHWE. This study is the first scientific report on the phytochemical composition of *V. fastigiata*, providing insight into the components of the chemo-diversity of this important plant.

## 1. Introduction

The genus *Vernonia*, belonging to the family Asteraceae, is made up of more than a thousand species distributed in the New and Old Worlds [1]. Many of these species, particularly *Vernonia amygdalina*, have been noted to be rich in nutrients [2] and possessing biological activities such as anti-bacterial [3], anti-viral [4] and anti-malarial/anti-plasmodial properties [5]. The bioactive properties of this species have been attributed to the presence of sesquiterpene lactones (SL) [3] and flavonoids [6]. *Vernonia fastigiata* Oliv. & Hiern is a perennial herb 0.1 to 1 m tall and is a basionym for *Parapolydora fastigiata* [7]. This plant has been said to possess anti-plasmodial [8] and anti-bacterial properties [9]. However, little is known about the phytochemical composition.

Traditionally, phytochemical studies often involve organic solvent-based extractions, however, these are quite toxic and have called for the development of safer alternative extraction methods [10]. Pressurized hot water extraction (PHWE) is a green extraction method that uses pressurized water at elevated temperatures to extract different classes of plant compounds. At these elevated temperatures and pressures, the polarity of water decreases close to that of common alcohols (e.g., methanol and ethanol). Thus, plant compounds of varying polarities can be dissolved and extracted by water under these conditions [10,11]. The ability of PHWE to extract different bioactive plant compounds has been shown elsewhere [12,13]. PHWE is a technique capable of extracting a plethora of plant metabolites and analysis is often accomplished by liquid chromatography hyphenated to mass spectrometry (LC-MS). To study the resulting high dimensional LC-MS data, visualization statistical models must be applied [14].

The current study was undertaken primarily to study the phytochemistry of *V. fastigiata* with the aid of the green extraction technology PHWE along with multivariate analysis methods. Here, principal component analysis (PCA) score plots were used to reduce the dimensionality of the data sets and to visualize the chemo-diversity of extracts achieved at various temperatures. Moreover, the interactive liquid/gas chromatography mass spectrometry (XCMS) cloud plots [15] provided statistical descriptions of qualitative and quantitative (temperature-related) changes in extracted metabolites.

## 2. Results and Discussion

Several *Vernonia* species are used for their nutritional value and as folklore remedies for the treatment of various human ailments, various compounds responsible for these nutraceutical and biological activities have been isolated and studied [2]. Although *V. fastigiata* has been found to possess anti-malarial [8] and anti-bacterial activities [9], the phytochemical composition of this plant has not been studied. Thus, in this study, we explored the phytochemical compounds of *V. fastigiata* using the green extraction technique PHWE, operating at different temperatures (50 °C, 100 °C, 150 °C and 200 °C).

**Figure 1:**
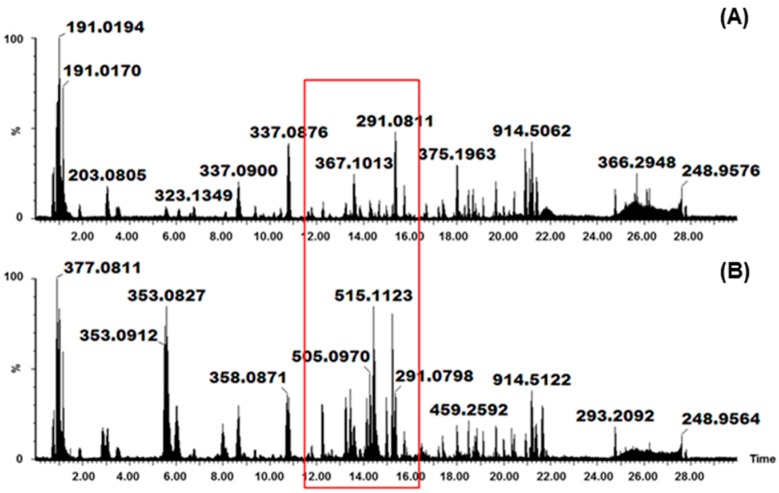
Ultra-high performance liquid chromatography (UHPLC) mass chromatograms, obtained using electrospray ionization (ESI) in negative mode, representing pressurized hot water extraction (PHWE) of *Vernonia fastigiata* metabolites at: (**A**) 50 °C; and (**B**) 200 °C.

Traditional medicine practitioners often boil or mix medicinal plants with warm water to achieve the extraction of different classes of bioactive compounds. Thus, PHWE is a feasible “green” extraction technique that mimics the extraction methods used by most traditional healers. This method of extraction is non-toxic and takes advantage of the changes in the physicochemical properties of water [10]. At a high temperature and constant pressure, the dielectric constant of water decreases and, consequently, at different temperatures, water is capable of extracting compounds with a wide polarity range, from polar to mid-polar to non-polar (Supplementary Materials, Figure S1). Furthermore, this is also shown in the ultra-high performance liquid chromatography-mass spectrometry (UHPLC-MS) chromatograms (Figure 1), where the greatest variation in peak intensity was seen between temperatures of 50 °C and 200 °C. From these results, the peak intensity of mid-polar and non-polar metabolites is low within the samples extracted at 50 °C in comparison to that extracted at 200 °C (Figure 1). At room temperature or low temperatures, water is highly polar (i.e., has a high dielectric constant) and viscous; however, an increase in temperature results in a decrease in these properties accompanied by an increase in the diffusive properties of water through the cell matrix [10]. Thus, at high temperature, water exhibits heightened extractive abilities of compounds with varying polarities [11]. However, MS-based methods are known to result in very high dimensional data which, sometimes, is difficult to handle with traditional statistics and, as such, multivariate data models are preferred to unearth such subtle differences [16].

**Figure 2:**
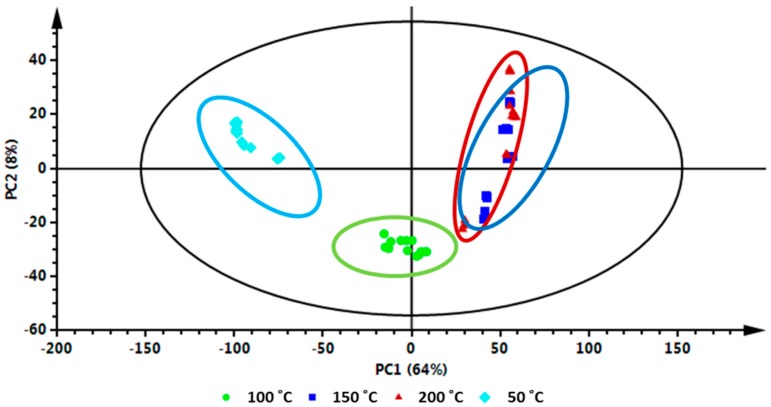
Principal component analysis (PCA) of the ultra-high performance liquid chromatography-mass spectrometry (UHPLC-MS) chromatograms from negative ionization data. The 2D score plot, explaining 72% of the total variation, shows differences in the clustering patterns of *V. fastigiata* samples extracted at different temperatures using PHWE.

The effect of temperature on the extraction of the bioactive compounds was consequently investigated using PCA as a multivariate statistical tool (Figure 2). This unsupervised chemometric model extracts meaningful information from high dimensional metabolic datasets and highlights similarities or differences within and between sample groupings [17]. The score plot depicted sample groupings (Figure 2), where all the samples representing extracts achieved at 50 °C and 100 °C, respectively, are separately clustered together, and also separated from the other samples (thus extracts achieved with 150 °C and 200 °C). Interestingly, samples extracted at 150 °C and 200 °C can be seen to form a very distinctive cluster, together and away from samples achieved at 50 and 100 °C. Moreover, the tight grouping of samples extracted at 150 °C and 200 °C is an indication that these samples share common metabolite profiles (Figure 2). In addition, the PCA score plot shows differences between groups along the x-axis and differences within groups along the y-axis with 72% of the total variation accounted for, showing the robustness of this model.

**Figure 3:**
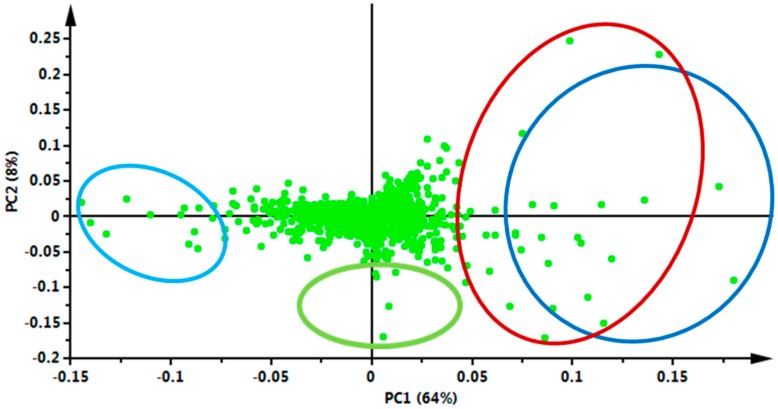
PCA loadings plot showing the selection of *V. fastigiata* metabolites associated with different extraction temperature conditions. The mass ions on the upper-left quadrant (light-blue ring) are associated with PHW extraction at 50 °C; ions on the lower-right quadrant (green ring) are associated with extraction at 100 °C, and ions on the right (upper and low; red and dark-blue ring) quadrants are associated with extraction at 150 °C and 200 °C.

**Figure 4:**
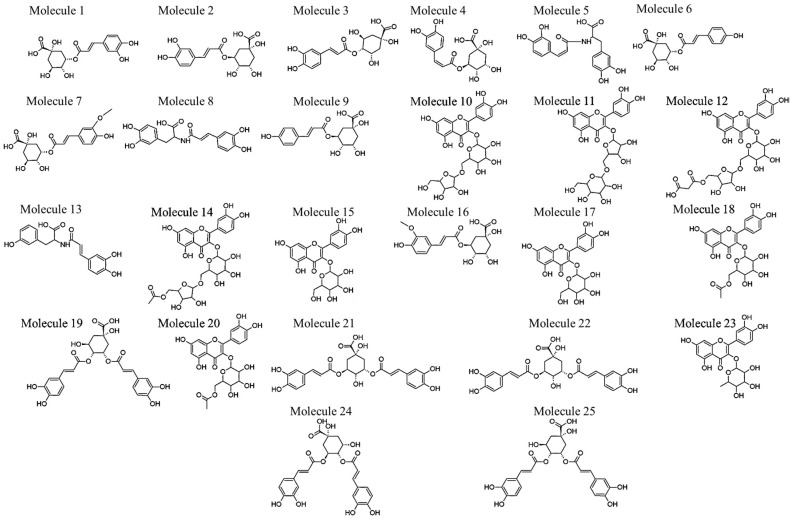
Chemical structures of annotated hydroxycinnamic acid (HCA) derivatives and quercetin-derived flavonoids in *V. fastigiata*.

From the PCA loadings plot (Figure 3), mass ions associated with the distribution patterns observed on the PCA score plot between groupings were further investigated. From these investigations, different metabolites were tentatively identified (Table 1, Figure 4) using metabolite information databases such as Dictionary of Natural Products (DNP) [18] and KNApSAck [19]. A total of 25 different metabolites made up of hydroxycinnamic acid (HCA) derivatives and quercetin-derived flavonoids were tentatively identified (Table 1, Figure 4).

**Figure 5:**
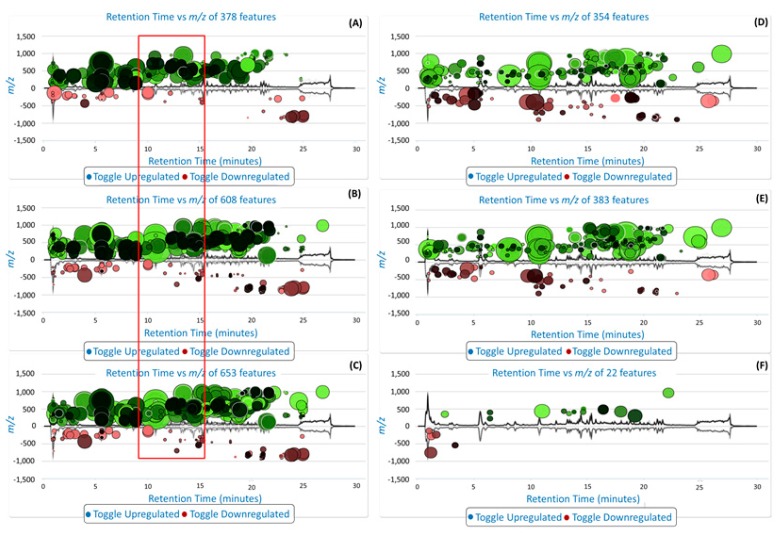
Liquid/gas chromatography mass spectrometry (XCMS) cloud plot of UHPLC-MS data: (**A**) ion comparisons between 50 °C and 100 °C; (**B**) ion comparisons between 50 °C and 150 °C; (**C**) ion comparisons between 50 °C and 200 °C; (**D**) ion comparisons between 100 °C and 150 °C; (**E**) ion comparisons between 100 and 200 °C; and (**F**) ion comparisons between 150 °C and 200 °C. The ions that are enhanced due to an increase in temperature (100 °C, 150 °C and 200 °C) are shown in the top plots (in green) and the ions that were extracted at a low temperature are shown in the bottom plot (in red).

To further complement the descriptive view provided by the PCA score plot (Figure 2) and to visualize the metabolite variation between samples extracted at different temperatures, XCMS online-based cloud plots [15] were constructed (Figure 5). The cloud plots show data characteristics such as *p*-value, log fold change, *m*/*z* ratio, Rt and signal intensity for each metabolite feature ion with a unique *m*/*z* ratio and Rt. From these cloud plots (Figure 5), a total of 378 features were detected between 50 °C and 100 °C (Figure 5A), 608 features were detected between 50 °C and 150 °C (Figure 5B) and 653 features were detected between 50 °C and 200 °C (Figure 5C), an indication that temperature did indeed affect the number of metabolites extracted. On the cloud plot, features with increased intensity due to the increase in extraction temperature (100 °C, 150 °C and 200 °C) are shown on top of the plot (in green) and features with low intensity due to low extraction temperature (i.e., 50 °C) are shown at the bottom of the plot (in red) (Figure 5). As stated above, the plots show that the number of features (phytochemicals) that were altered/extracted are associated with an increase in temperature. In addition, these plots show an increase in temperature results in an increase in the extraction of mid-polar (red box) and non-polar phytochemicals. This observation reiterates the role of the extraction temperature for efficient and selective extraction of metabolites during PHWE.

**Figure 6:**
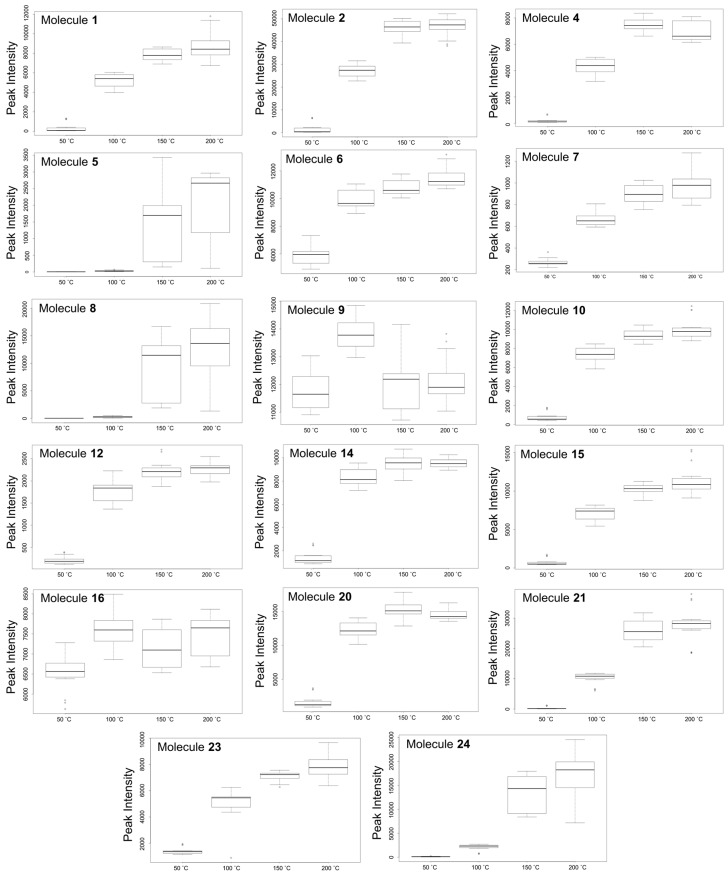
Box-and-whiskers plots showing the optimal temperature conditions for 17 different metabolites indicated in Table 1. All significant differences are based on ANOVA with *p* ≤ 0.01. Metabolites are made up of hydroxycinnamate derivatives (**1**, **2**, **4**–**9**, **16**, **21** and **24**) and quercetin-derived flavonoids (**10**, **12**, **14**, **15**, **20** and **23**) extracted from *V. fastigiata* during PHW extraction.

The results show a further increase in temperature, however, leads to a point of saturation where the number of extracted metabolites do not increase beyond a certain extraction temperature (Figure 5F), i.e., between 150 °C and 200 °C in this case. Here, only 22 features were altered between 150 and 200 °C (Figure 5F), compared to the 653 features that were altered between 50 °C and 200 °C (Figure 5C). This cloud plot (Figure 5F) explains the clustering of plant samples extracted at these temperatures (150 °C and 200 °C) previously observed on the PCA score plot (Figure 2), where very little differences between these samples were observed. Furthermore, at such elevated temperatures (150 °C and 200 °C) differences in the classes of extracted metabolites may be too minor to show significant differences or thermally labile metabolites may have undergone degradation [10]. For instance, previously it was reported that elevated temperatures resulted in the formation of carcinogenic compounds such as hydroxymethylfurfural [30]. These possibilities suggest that some metabolites may require optimal temperature conditions for extraction. The best extraction temperature condition(s) for each annotated metabolite (Table 1) were graphically described by box-and-whiskers plots (boxplot) (Figure 6), where, following ANOVA analysis, it was possible to indicate temperature conditions that resulted in significant differences (*p* value ≤ 0.01) in yield patterns for each metabolite. However, the levels of some pharmacologically relevant metabolites (e.g., **3**, **11**, **13**, **17**–**19**, **22** and **25**) were not statistically significant (*p* value ≤ 0.01) across data sets and were thus not graphically presented by boxplots. From the boxplots (Figure 6), the general trend observed is that an increase in temperature results in an increase in the intensity of each molecule/metabolite. However, sensitivity to temperature of some molecules was also observed, where at temperatures below 100 °C, molecules **5** and **8** were not extracted at all. However, the intensities of these molecules show an increase with an increase in temperature, for instance at 150 °C and 200 °C. Some molecules, however, were negatively affected by temperatures beyond 100 °C and show a decrease in the intensity, possibly due to thermal decomposition (for instance, molecule/metabolite **9**) (Figure 6).

In Table 1, different metabolites were tentatively identified using DNP [18] and KNApSAcK [19] metabolite information databases. Authentic standards were also used to identify some metabolites, however, due to the lack of standards for the rest of the metabolites, MS fragmentation patterns and chromatographic elution order for flavonoids [21,26,27,28] and chlorogenic acids [20,22,23] were compared with literature reports for annotation. Table 1 shows that the leaves of *V. fastigiata* are rich in chlorogenic acids (CGAs) as well as in glycosylated quercetin derivatives (flavonols). Interestingly, other biologically active HCA derivatives, namely clovamide (**5** and **8**) [24,31] and deoxyclovamide (**13**) [31], were also found.

### 2.1. Identification of Various HCA Derivatives in V. fastigiata

HCA derivatives found in *V. fastigiata* include **1**–**9**, **13**, **16**, **19**, **21**–**22** and **24**–**25** (Table 1 and Figure 4). For annotated chlorogenic acids (**1**–**4**, **6**–**7**, **9**, **16**, **19**, **21**–**22** and **24**–**25**), six types of fragmentation rules were noted: neutral ions attributed to the loss of a quinic acid (−174 Da), caffeic acid (−162 Da), *p*-coumaric acid (−146 Da) and ferulic acid moieties (−176 Da), as well as carbon dioxide (−44 Da) and H_2_O (−18 Da).

Molecules **1**–**4** with a precursor ion, [M − H]^−^, at *m*/*z* 353 were identified as mono-caffeoylquinic acids. Further inspection of the UHPLC chromatogram and mass spectra of these metabolites revealed **1** at Rt 2.87 min to contain fragment ions at *m*/*z* 191, 179 and 135. These ions resulted from the loss of a caffeic acid moiety, the loss of a quinic acid moiety and the decarboxylation of the caffeic acid moiety, respectively. The relative abundance of the ion at *m*/*z* 191 base peak (bp) relative to the ion at *m*/*z* 179 is a typical characteristic of 3-caffeoylquinic acid (3-CQA) [20]. For **2** at Rt 5.56 min, a single fragment ion at *m*/*z* 191 was obtained due to the sequential loss of a caffeic acid moiety and **2** was characterized as 5-CQA [20,21]. Molecule **3** at Rt 6.00 min showed fragment ions at *m*/*z* 191, 179 and 173 resulting from the loss of a caffeic acid moiety, loss of a quinic acid moiety and the presence of a dehydrated quinic acid moiety, respectively. The presence of the ion at *m*/*z* 173 is diagnostic of a HCA derivative esterified at position 4 on the quinic acid [22] thus **3** was annotated as 4-CQA. Molecule **4** at Rt 7.99 min showed the same fragmentation pattern as **2**, thus this molecule was annotated as an isomer of 5-CQA. Based on the elution order suggested by Clifford et al. on a reverse-phase column, **4** relative to **2** was annotated as *cis*-5-CQA [23]. Geometrical isomers of HCA derivatives have been known to show the same MS fragmentation patterns [23,32] and the ability to distinguish *cis* isomers from their *trans* counterparts has proven to be a challenge.

However, progress has been made where *cis* isomers of di-caffeoylquinic acids (diCQA) were shown to preferentially bind to alkali metals, whereas *trans*-isomers did not [32]. In addition, positional isomers of mono-CQAs in this study were identified with the use of authentic standards.

Molecule **5** and **8**, at Rt 8.14 min and 10.74 min, respectively, showed a precursor ion at *m*/*z* 358 [M − H]^−^ (Supplementary Materials, Figure S2). For both molecules (**5** and **8**), the fragment ions observed were 222, 178 and 161 (Supplementary Materials, Figure S3) due to the loss of the vinyl-catecholic fragment [M − H − 136]^−^, the loss of a deaminated l-DOPA (3,4-dihydroxyphenylalanine) fragment [M − H − 180]^−^ [24] and the ion at *m*/*z* 161 possibly representing a dehydrated-caffeoyl amide moiety. Thus, **5** and **8** were characterized as clovamide (*N*-caffeoyl-l-DOPA (3,4-dihydroxyphenylalanine)) isomers. From a study by Locatelli et al., where the *trans* and *cis* isomers of clovamide were noted, the earlier eluting isomer (thus **5**) was identified as the *cis* isomer and the later eluting isomer (thus **8**) was identified as the *trans* isomer [25]. Molecules **6** and **9** showed a precursor ion of *m*/*z* 337 [M − H]^−^ at Rt 8.64 and 10.82 min, respectively. Molecule **6** showed fragment ions at *m*/*z* 191 and 163, representing the loss of both *p*-coumaroyl and quinic acid moieties, respectively. The abundance of the ion at *m*/*z* 191 relative to the ion at *m*/*z* 163 characterized **6** as 3-*p*-coumaroylquinic acid (3-*p*CQA). Molecule **9** showed a single fragment ion at *m*/*z* 191 resulting from the loss of a *p*-coumaroyl moiety, thus **9** was annotated as 5-*p*CQA [22]. Molecules **7** and **16** at Rt 10.13 min and 13.60 min, respectively, showed a precursor ion at *m*/*z* 367 [M − H]^−^. Molecules **7** and **16** showed fragment ions at *m*/*z* 191 and 193 representing the loss of a feruloyl and quinic acid moieties, respectively. The abundance of the ion at *m*/*z* 191 led to the annotation of **7** as 3-feruloylquinic acid (3-FQA) [20]. The presence and abundance of fragment ion at *m*/*z* 191 supported the annotation of **16** as 5-FQA [20].

Molecule **13** at Rt 12.81 min showed a precursor ion at *m*/*z* 342 and a fragment ion at *m*/*z* 206 (Supplementary Materials, Figure S4) due to the loss of the vinyl-catecholic fragment [M − H − 136]^−^. The molecular formula (C_18_H_17_NO_6_) of this compound led to the tentative annotation as deoxyclovamide (*N*-caffeoyl-l-tyrosine). Molecules **19**, **21**, **22**, **24** and **25** showed precursor ions at *m*/*z* 515 [M − H]^−^, suggesting di-caffeoylquinic acid (diCQA) isomers. Molecules **19**, **24** and **25** showed fragment ions at *m*/*z* 173, which, as stated above, is indicative of acylation at position 4 on the quinic acid unit. Molecules **19** and **25** at Rt 14.13 and 16.49 min, respectively, showed similar fragmentation patterns. The observed fragment ions were at *m*/*z* 335, 191, 179 and 173, thus **19** and **25** were annotated as 3,4-diCQA isomers. As the plant biosynthetic pathway produces molecules in the *trans* geometry and exposure of the plant to the suns UV-rays causes photochemical isomerization to the *cis* geometry [23], the low peak intensity of **19** relative to **25** suggests that the former is a *cis* isomer of the latter.

Molecule **24** at Rt 15.24 min was annotated as 4,5-diCQA due to the absence of the fragment ion at *m*/*z* 335, the presence of the fragment ions at *m*/*z* 179, 191 and abundance of the ion at *m*/*z* 173. Molecules **21** and **22** at Rt 14.42 and 14.58 min, respectively, were annotated as 3,5-diCQA isomers due to the presence of ions at *m*/*z* 191 and 179, the abundance of the fragment ion at *m*/*z* 191 and the absence of the ion at *m*/*z* 173. Due to the high peak intensity of **21** relative to **22**, it is suggested that the latter is the *cis* isomer of the former.

#### Biological Relevance of Identified HCA Derivatives

Plant secondary metabolites such as CGAs are HCA derivatives and are formed from the transesterification reaction between a quinic acid unit and HCAs such caffeic acid, ferulic acid and *p*-coumaric acid [22,23]. The *trans*-HCA can be esterified to one or more of the four hydroxyl groups at positions 1, 3, 4 and 5 on the quinic acid unit, thus giving rise to various positional isomers. Therefore, from Table 1, *V. fastigiata* contains a number of both mono- and di-acylated CGAs (**1**–**4**, **6**–**7**, **9**, **16**, **19**, **21**–**22**, and **24**–**25**). These metabolites and their derivatives have also been isolated and identified in other *Vernonia* species [1,2]. Moreover, these compounds have been associated with various health benefits and are acclaimed for their anti-oxidant activity [33]. The caffeic acid-derived CGAs show the most potent anti-oxidant activity, which is attributed to the catechol group at positions 3 and 4 on the HCA [21]. In some plants, of the four positional isomers (1, 3, 4 and 5-acylated isomers), 5-caffeoylquinic acid (5-CQA) is considered a major anti-oxidant [34]. In addition, other positional isomers have been noted to possess the ability to prevent liver disease (for instance, 3-CQA) [35] and possess anti-inflammatory activity (for instance, 4-CQA) [36]. The presence of diCQAs were also annotated in *V. fastigiata*, where in another study 3,4-diCQA and 3,5-diCQA were noted to possess anti-microbial and anti-viral activities [37] with 3,5-diCQA showing anti-human immunodeficiency virus (HIV) type 1 DNA integrase activity [38]. Furthermore, 4,5-diCQA has been noted to exhibit anti-inflammatory effects [39]. In addition to possessing positional isomers of CGAs (i.e., **1**–**3**), *V. fastigiata* also contains geometrical isomers of some of these phenolic compounds, for example 5-CQA (**2**) and *cis*-5-CQA (**4**). This large pool of (poly)phenolics is described by the “better safe than sorry” phenomenon [21]. Whereby, for survival and protection, the plant produces multiple structurally similar metabolites that contribute to the state of readiness within the plant. As sessile organisms, plants are constantly exposed to various stressors resulting in oxidative stress, thus to mitigate the effects thereof plants use the readily available phenolic compounds of similar structures [21]. Therefore, the similarities and minor differences in structural configuration of the phenolic compounds can be thought to add to the diversity of the plant metabolome.

Furthermore, two other HCA-derived metabolites; clovamide (**5** and **8**) (*N*-caffeoyl-l-DOPA) and deoxyclovamide (**13**) (*N*-caffeoyl-l-tyrosine), were also extracted. Clovamide is a bioactive compound and reported to possess radical scavenging properties [24]. To the best of our knowledge this is the first identification of this metabolite in Old World *Vernonia* species such as *V. fastigiata* [1,7]. Clovamide was, however, identified by Martucci et al. in New World *Vernonia* species found in the genera *Chrysolaena* (*Vernonia herbacea* and *Vernonia platensis*) [1]. These two species were the only two of the ten *Vernonia* species studied that accumulated this metabolite, and thus clovamide was used as a chemo-marker for the taxonomic classification of the species found in the *Chrysolaena* genera. Clovamide has been noted as being abundant in cacoa seeds from which cocoa beverages and chocolates are produced [40]. This metabolite is structurally similar to rosmarinic acid (also a caffeic acid derivative), a known potent anti-oxidant, and the two have been shown to have comparative anti-oxidant properties [41]. In addition, clovamide exhibited the inhibition of lipid peroxidation [25] and in a separate study by Fallarini et al., was found to have neuroprotective effects [42]. Deoxyclovamide as an anti-oxidant has also been reported elsewhere, although showing less anti-oxidant activity than clovamide [31].

### 2.2. Identification of Various Glycosylated Quercetin Derivatives in V. fastigiata

*V. fastigiata* leaves contain a variety of glycosylated quercetin metabolites, where **10**–**12**, **14**–**15**, **17**–**18**, **20** and **23** were identified as glycosylated quercetin derivatives due to the presence of the fragment ion at *m*/*z* 301. Characterization was achieved by the loss of neutral ions attributed to hexose (−162 Da), pentose (−132 Da), rhamnose (−146 Da), disaccharide of glucose and pentose (−294 Da), malonyl pentose (−218 Da), malonyl (−86 Da) or acetyl moieties (−42 Da).

Molecules **10** and **11** with a [M – H]^−^ ion at *m*/*z* 595 were characterized as quercetin-hexoside-pentoside and quercetin-pentoside-hexoside, respectively [26,27]. For **10** at Rt 12.23 min, fragment ions at *m*/*z* 463, 301 and 300 were obtained due to the sequential loss of a terminal pentose and hexose moieties [26]. Molecule **11** at Rt 12.41 min showed fragment ions at *m*/*z* 433, 301 and 300, and these resulted from the sequential loss of a terminal hexose and pentose moieties [27]. Due to the abundance of the radical aglycone ion at *m*/*z* 300 relative to the aglycone ion at *m*/*z* 301, glycosylation of quercetin was determined to be at position 3 for both molecules [29]. Thus, **10** was characterized as quercetin-3-*O*-hexoside-*O*-pentoside and **11** as quercetin-3-*O*-pentoside-*O*-hexoside.

Molecule **12** at Rt 12.64 min showed a precursor ion at *m*/*z* 681 [M − H]^−^ as well as fragment ions at 595, 463, 301 and 300 resulting from the sequential loss of the malonyl, pentose and hexose moieties, respectively. As such, due to the abundance of the radical aglycone at *m*/*z* 300, **12** was characterized as quercetin-3-*O*-hexoside-*O*-pentoside-*O*-malonyl. The mass spectra of **14** at Rt 13.24 min displayed a precursor ion at *m*/*z* 637 [M − H]^−^ and three fragment ions with one at *m*/*z* 595 due to the loss of a terminal acetyl moiety, one at *m*/*z* 463 due to the loss of a pentose moiety and one at *m*/*z* 301 due to the subsequent loss of a hexose moiety. Molecule **14** was thus characterized as quercetin-3-*O*-hexoside-*O*-pentoside-*O*-acetyl due to the observed abundance of the radical aglycone ion at *m*/*z* 300. Molecule **15** and **17** showed a precursor ion at *m*/*z* 463 [M − H]^−^ with Rt 13.42 and 13.71 min, respectively. Mass spectra of these two molecules showed fragment ions at *m*/*z* of 301 and 300, due to the loss of a terminal hexose moiety with *m*/*z* 162. The abundance of the radical aglycone ion at *m*/*z* 300 indicated that the glycosylation of quercetin was at position 3. Thus **15** and **17** were characterized as quercetin-3-*O*-hexoside isomers. However, considering the elution order of the two molecules on a reverse-phase column, **15** was characterized as quercetin-3-*O*-galactoside and **17** as quercetin-3-*O*-glucoside [26,28].

Molecules **18** and **20** at Rt 14.04 min and 14.28 min, respectively, showed a precursor ion at *m*/*z* 505 [M − H]^−^. For both molecules (**18** and **20**), the fragment ions observed were 463, 301 and 300, and resulted from the loss of the terminal acetyl and hexose moieties. Thus **18** and **20** were characterized as quercetin-3-*O*-hexoside-acetyl isomers, because of the relative abundance of the radical aglycone ion at *m*/*z* 300. Considering the elution order, these metabolites were characterized as quercetin-3-*O*-galactoside-acetyl (**18**) and quercetin-3-*O*-glucoside-acetyl (**20**). Molecule **23** was characterized as quercetin-rhamnoside with a precursor ion at *m*/*z* 447 [M − H]^−^ and Rt at 15.00 min, due to the presence of the fragment ions at *m*/*z* 301 and 300, resulting from the loss of a rhamnose moiety [28]. The abundance of the fragment ion at *m*/*z* 300 indicated glycosylation at position 3, thus **23** was characterized as quercetin-3-*O*-rhamnoside.

#### Biological Relevance of Identified Quercetin Glycosylated Derivatives

Flavonoids are a major class of secondary metabolites in plants, and these polyphenolic compounds are ubiquitous and involved in providing plants with their rich colors [43]. Flavonoids also act as secondary protectants for plants against different stressors, such as microbial infections or UV-B radiation [21,43]. However, the upsurge in the interest of flavonoids is due to the proposed health benefits, particularly the anti-oxidant activity. Of the many flavonoid classes that exist, the bioactivity of the flavonol quercetin is well documented and is considered to be one of the most potent of flavonoid anti-oxidants, where the anti-oxidant activity is dependent on the hydroxyl substitution pattern on the B-ring [43]. Similar to caffeoyl-containing phytochemicals, the 3′,4′-catechol unit found on the B-ring makes quercetin a significant scavenger of reactive oxygen species (ROS) [21]. Another mechanism of anti-oxidant action used by quercetin is the suppression of ROS formation by chelating free metal ions that enhance the process. Here the catechol unit along with other specific positions (3-OH, 4-oxo and 5-OH) on the different rings, enhances the metal-chelating and metal-stabilizing properties [43].

The aglycone, quercetin, has lipophilic properties and glycosylation enhances the solubility thereof in the plant, thus allowing easier transportation into various cellular compartments. Glycosylation or any modification of the aglycone reduces the anti-oxidant activity of the metabolite, compared to the unbound aglycone, as specific positions (3-OH or 4′-OH) involved in radical scavenging and/or ion-suppression may be occupied by sugar moieties. However, glycosylation is significant for the bioavailability of the aglycone in plasma [43], and following hydrolysis of the sugar moiety, the aglycone is absorbed and found in the plasma where it can exhibit different biological activities. Moreover, quercetin has growth inhibitory effects against several malignant tumor cell lines [43]. Lastly, in an interesting study by Ganesh et al., quercetin and related quercetin derivatives were found to exhibit anti-plasmodial activity [44], which might explain the purported anti-plasmodial activity of *V. fastigiata* reported by Clarkson et al. [8].

## 3. Materials and Methods

### 3.1. Materials

Authentic standards of caffeic acid-derived chlorogenic acids (3-caffeoylquinic acid; 4-caffeoylquinic acid; 5-caffeoylquinic acid; 1,3-di-caffeoylquinic acid; 1,5-di-caffeoylquinic acid; 3,4-di-caffeoylquinic acid; 3,5-di-caffeoylquinic acid and 4,5-di-caffeoylquinic acid) and flavonols (quercetin-3-*O*-glycoside; quercetin-4-*O*-glycoside; quercetin-7-*O*-glycoside and rutin) were purchased from Phytolab (Vestenbergsgreuth, Germany). Analytical-grade acetonitrile was obtained from Romil (Cambridge, UK). Formic acid and diatomaceous earth were obtained from Sigma-Aldrich (St. Louis, MO, USA).

### 3.2. Methods

#### 3.2.1. Extraction

*V. fastigiata* leaves were collected from the Venda region of the Limpopo province (South Africa). Herbarium specimens (with voucher number MN 027/2016) were prepared and stored in the Department of Botany at the University of Venda. Sample preparation and extraction procedures were conducted according to Khoza et al. [13]. Briefly, *V. fastigiata* leaves were air-dried and pulverized using a mortar and pestle. These dried, ground leaves (3 g) were then mixed with diatomaceous earth (1 g) and metabolites were extracted with pure water (pH 6.8) using a homemade PHWE system [12]. The instrument was set at the desired temperature levels (50 °C, 100 °C, 150 °C and 200 °C), at a constant pressure of 1000 ± 100 psi (68.9 ± 6.89) and flow rate of 5 mL/min. Five samples (50 mL) per temperature setting were collected. Two milliliters (2 mL) of the extracts were filtered through 0.22 μm membrane syringe filters into glass vials and stored at 4 °C prior to ultra-high performance liquid chromatography-quadrupole time of flight mass spectrometry (UHPLC-qTOF-MS) analysis.

#### 3.2.2. Chromatography and Mass Spectrometry Analyses

Extracts obtained from the PHWE method were analyzed by injecting 3 µL into a Synapt G1 UHPLC-high definition MS instrument (Waters Corporation, Manchester, UK) equipped with a Waters Acquity HSS T3 C18 column (150 mm × 2.1 mm with particle size of 1.8 μm), analyzed at a flow rate of 0.4 mL/min. Three technical replicates from five independent extracts per temperature point were analyzed on the UHPLC-qTOF-MS, resulting in fifteen replicates per temperature point. The mobile phase for the chromatographic analysis consisted of 0.1% formic acid in MilliQ water (solvent A) and 0.1% formic acid in acetonitrile (solvent B). The initial conditions were 2% B at 1 min, followed by multiple gradients to 60% B at 24 min and a steep gradient to 95% B at 25 min, the conditions were kept isocratic for 2 min and then dropped to the initial conditions in 1 min, followed by 2 min isocratic wash at 2% B to re-equilibrate the column. The column temperature was maintained at 60 °C. This 30 min chromatographic elution was monitored by both photodiode array (PDA) and MS detectors. The PDA detectors (Waters Corporation, Manchester, UK) scanning range was set from 200 nm to 500 nm, with 1.2 nm bandwidth resolution and a sampling rate of 20 points/s. For MS, data were acquired in negative and positive modes using optimized settings presented elsewhere [13], with the scan time set at 0.1 s. In addition, increasing collision energies (CE) (10 eV, 20 eV, and 40 eV) were utilized to achieve useful MS fragmentation data, for identification purposes.

#### 3.2.3. Data Processing and Multivariate Data Analysis (MVDA)

UHPLC-MS raw data (ESI-negative mode) were extracted and imported into MassLynx^TM^ XS software (SCN 704, Waters Corporation, Mildford, MA, USA) for data processing. Briefly, the following parameters were chosen: chromatogram retention time (Rt) range of 5–23 min, mass range of 100–1000 Da, mass tolerance of 0.02 Da, and Rt window of 0.2 min. The data matrix from MarkerLynx was then exported into SIMCA version 13.0 software (Umetrics, Umea, Sweden) for PCA analyses. Before PCA modelling, the pre-processed X data were centered and Pareto-scaled to put all variables on equal footing, minimize variable redundancy and adjust for measurement errors. Furthermore, a nonlinear iterative partial least squares algorithm (in-built within SIMCA) was used to handle the missing values, with a correction factor of 3.0 and a default threshold of 50%. A seven-fold cross-validation (CV) method was applied as a tuning procedure in computing the PCA models. To complement SIMCA-based chemometric analyses, the raw data were further analyzed using cloud-based informatics software (XCMS Online) [15].

XCMS online software is an open-source web-based software developed to process and visualize untargeted MS-based metabolomics data [14]. The enhanced online interface allowed two-group (paired) comparisons between different temperature conditions (i.e., 50 °C vs. 100 °C and 50 °C vs. 150 °C etc.). In addition, multi-group comparisons between the four temperature conditions (50 °C, 100 °C, 150 °C and 200 °C) were also performed. These comparisons (paired and multi-group) were monitored with the interactive visualization tool known as a cloud plot [14]. Following the uploading of the raw data files (NetCDF), parameters for XCMS processing were as follows: matchedFilter for feature detection (with 0.1 step size to use for profile generation and 30 full width at half maximum matched filtration Gaussian model peak), and peak groups for Rt correction (with linear alignment, 1 extra peak allowed in Rt correction groups and only 1 number of missing samples allowed in Rt correction groups). The chromatogram alignment parameters were: minifrac of 0.5 (minimum fraction of samples necessary in at least one of the sample groups for it to be a valid group), bandwidth (bw) of 10 s (allowable Rt deviations) and *m*/*z* width of 0.25 (width of overlapping *m*/*z* slices to use for peak density chromatograms and grouping peaks across samples). Each cloud plot showed a fold change of >1.5 and *p* values < 0.01, where the size of each bubble is associated with the log fold change of each feature [14]. The statistical significance of the fold change is represented by the color intensity of the feature, where features with darker colors have a higher *p* value than brighter colored features. The statistical significance of the fold change was calculated by a Welch *t* test with unequal variance for paired groups and calculated by ANOVA for multi-group comparisons. In addition, features denoted with a black outline are the ones with database hits in METLIN [45] and those denoted with a white outline are the ones with no METLIN-based hits.

The effect of temperature on extraction for each metabolite (feature) was visualized on box-and-whiskers plots (boxplots). Thus, the XCMS online analyses of the UHPLC-MS data were coupled with the MarkerLynx and SIMCA-based analyses to comprehensively study the role temperature plays during metabolite extraction by PHWE.

#### 3.2.4. Metabolite Annotation

From the PCA loadings plots, metabolites of which the levels were affected by the extraction temperature, were selected. Using the *m*/*z* of these metabolites, specific MS spectra were obtained and various fragmentation patterns were observed. These spectra were used to identify the various ions, and the identities of the ions were searched for and confirmed using online databases such as Dictionary of Natural Products (DNP) [18] and KNApSAck [19]. For further structural confirmation, MS data obtained with different collision energies (CE) were used to generate mass spectra containing detailed fragmentation patterns of the biological markers.

## 4. Conclusions

The results of the current study successfully demonstrated PHWE as a feasible “greener” extraction method of nutraceuticals from the medicinal plant *V. fastigiata*. The use of UHPLC-MS in combination with cloud plots was employed for the first time to statistically optimize and verify the pharmacological content from the PHWE extracts. Here, XCMS-based cloud plots were assessed in conjunction with PCA score plot to evaluate the nature of metabolites of which the levels were affected by variations in temperature for extraction. This use of the cloud plot allowed the visualization of metabolites of varying polarities that were affected by temperature on different reverse-phase chromatographic regions on the UHPLC-MS chromatograms.

This study is also the first scientific report that describes the diverse phytochemicals of *V. fastigiata*, linking the presence of HCA-derivatives (e.g., CGAs, clovamide and deoxyclovamide) and quercetin-derived flavonoids to its use as a medicinal plant. The presence of these phytochemicals promotes the use of *V. fastigiata* for various pharmacological activities as discussed. To the best of our knowledge this is the first report on the identification of clovamide and deoxyclovamide in an Old World *Vernonia* species. Previously, Martucci et al. identified clovamide as a marker for the chemotaxonomic classification of studied New World *Vernonia* species [1]. Such findings, with the presence of clovamide in an Old World *Vernonia* species, may call for the study of other chemotaxonomic markers linking Old World and New World *Vernonia* species.

## Figures and Tables

**Table 1:** Table listing annotated metabolites extracted from *Vernonia fastigiata* by pressurized hot water extraction (PHWE).

Molecule Number	Rt (min)	Compound Name	Negative Ionization (*m*/*z*)	Negative Ionization MS/MS	Reference
1	2.87	3-*O*-caffeoylquinic acid *	353.0860	353→191 **^#^**, 179,135	[20]
2	5.56	5-*O*-caffeoylquinic acid *	353.0887	353→191 **^#^**	[20,21]
3	6.00	4-*O*-caffeoylquinic acid *	353.0825	353→191, 179, 173 **^#^**	[20,22]
4	7.99	*cis*-5-*O*-caffeoylquinic acid	353.0806	353→191 **^#^**	[20,23]
5	8.14	*cis-N*-caffeoyl-l-DOPA (*cis*-clovamide)	358.0879	358→222, 178 **^#^**, 161	[24,25]
6	8.64	3*-O*-*p*-coumaroylquinic acid	337.0873	337→191 **^#^**, 163	[20,22]
7	10.13	3*-O*-feruloylquinic acid	367.1044	367→191 **^#^**, 193	[20]
8	10.74	*trans*-*N*-caffeoyl-l-DOPA (clovamide)	358.0871	358→222, 178 **^#^**, 161	[24,25]
9	10.82	5-*O*-*p*-coumaroylquinic acid	337.0901	337→191 **^#^**	[20,22]
10	12.23	quercetin-3-*O*-hexoside-*O*-pentoside	595.1245	595→301, 300 **^#^**, 271, 255, 243	[26]
11	12.41	quercetin-3-*O*-pentoside-*O*-hexoside	595.1288	595→301, 300 **^#^**, 271, 255	[27]
12	12.64	quercetin-3-*O*-hexoside-*O*-pentoside-*O*-malonyl	681.1300	681→637, 595, 301, 300 **^#^**, 271, 255	MS/MS spectra
13	12.81	*N*-caffeoyl-l-tyrosine (deoxyclovamide)	342.0944	342→342 **^#^**, 206	MS/MS spectra
14	13.24	quercetin-3-*O-*hexoside-*O*-pentoside-*O*-acetyl	637.1335	637→595, 301, 300 **^#^**, 271, 255, 243	MS/MS spectra
15	13.42	quercetin-3-*O*-galactoside	463.0857 ^#^	463→301, 300, 271, 255, 243	[26,28,29]
16	13.60	5-*O*-feruloylquinic acid	367.1008 ^#^	367→191	[20]
17	13.71	quercetin-3-*O*-glucoside (isoquercetin) *	463.0835	463→301, 300 **^#^**, 271, 255, 243	[26,28,29]
18	14.04	quercetin-3-*O*-galactoside-*O*-acetyl	505.0955	505→463, 301, 300 **^#^**, 271, 255, 243	MS/MS spectra
19	14.13	3,4-di-*O*-caffeoylquinic acid isomer	515.1113	515→353, 335, 191, 179, 173 **^#^**	[20,22]
20	14.28	quercetin-3-*O*-glucoside-*O*-acetyl	505.0970 ^#^	505→463, 301, 300, 271, 255	MS/MS spectra
21	14.42	3,5-di-*O*-caffeoylquinic acid isomer *	515.1123	515→353, 191 **^#^**, 179	[20,22]
22	14.58	3,5-di-*O*-caffeoylquinic acid isomer	515.1082	515→353, 191 **^#^**, 179	[20,22]
23	15.00	quercetin-3-*O*-rhamnoside (quercetrin)	447.0850	447→301, 300 **^#^**, 271, 255, 243	[28,29]
24	15.24	4,5-di-*O*-caffeoylquinic acid *	515.1099	515→353, 191, 179, 173 **^#^**	[20,22]
25	16.49	3,4-di-*O*-caffeoylquinic acid isomer *	515.1128	515→353, 335, 191, 179, 173 **^#^**	[20,22]

**^#^** base peak; * Metabolites with identical Rt and MS/MS spectra to available standards.

## Supplementary Materials

Supplementary Materials are available online. Figure S1: UHPLC ESI (neg) mass chromatograms obtained from PHW extraction of *V. fastigiata* metabolites at: (**A**) 50 °C; (**B**) 100 °C; (**C**) 150 °C; and (**D**) 200 °C. Figure S2: Extracted ion chromatograms of: (**A**) *cis*-clovamide; and (**B**) *trans*-clovamide. Figure S3: Mass spectra showing the fragmentation pattern for both *cis*- and *trans*-clovamide. Figure S4: Mass spectra showing the fragmentation pattern for deoxyclovamide.
