# Supplementary material for: A Metabolomics-Guided Exploration of the Phytochemical Constituents of Vernonia fastigiata with the Aid of Pressurized Hot Water Extraction and Liquid Chromatography-Mass Spectrometry
Source: Molecules. 2017 Jul 27;22(8):1200. doi: 10.3390/molecules22081200 (PMC6152066; doi:10.3390/molecules22081200)
Supplement: Supplementary file 1 [file molecules-22-01200-s001.pdf]

Supplementary data

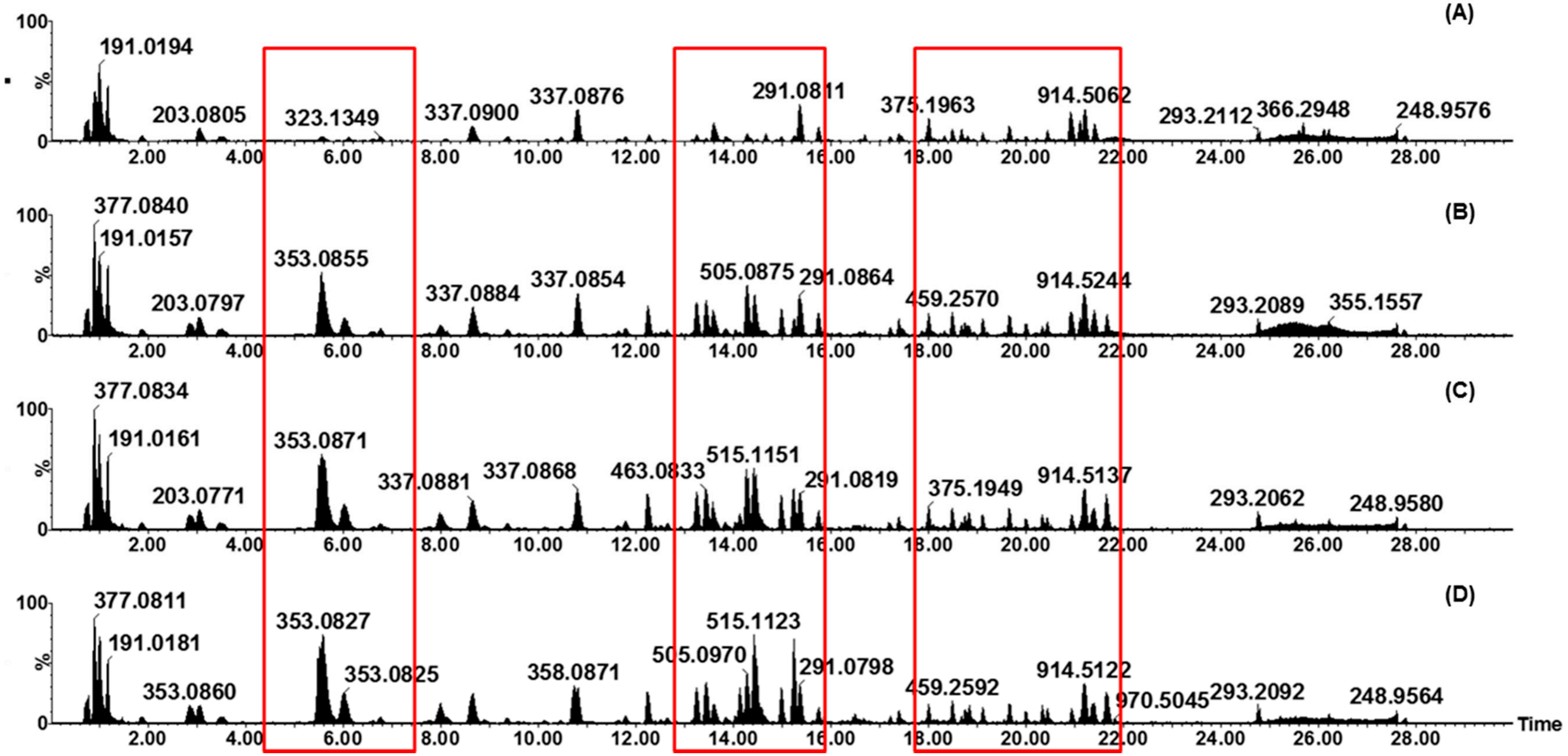

Figure S1. Ultra-high performance liquid chromatography (UHPLC) mass chromatograms, obtained using electrospray ionization (ESI) in negative mode, representing pressurized hot water extraction (PHWE) of *Vernonia fastigiata* metabolites at: (**A**) 50 ˚C, (**B**) 100 ˚C, (**C**) 150 ˚C and (**D**) 200 ˚C.

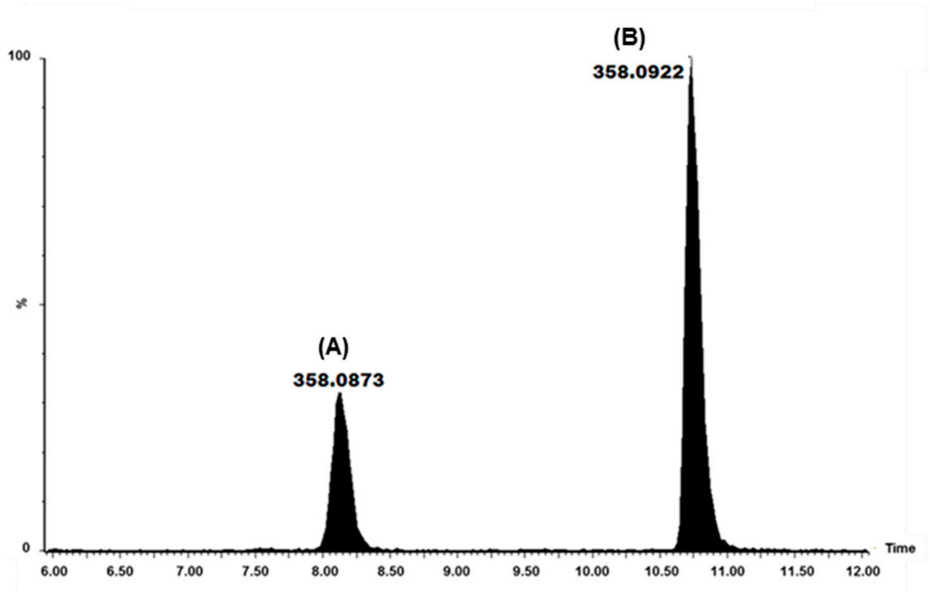

Figure S2. Extracted ion chromatograms of: (**A**) *cis*-clovamide and (**B**) *trans*-clovamide.

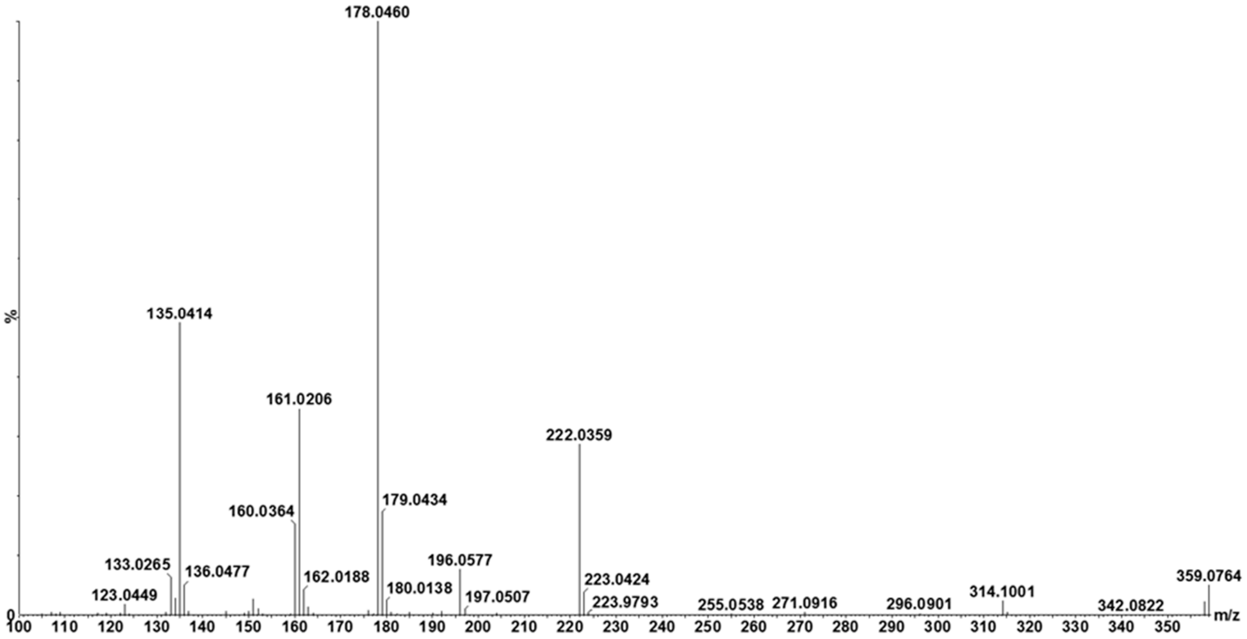

Figure S3. Mass spectra showing the fragmentation pattern for both *cis*- and *trans*-clovamide.

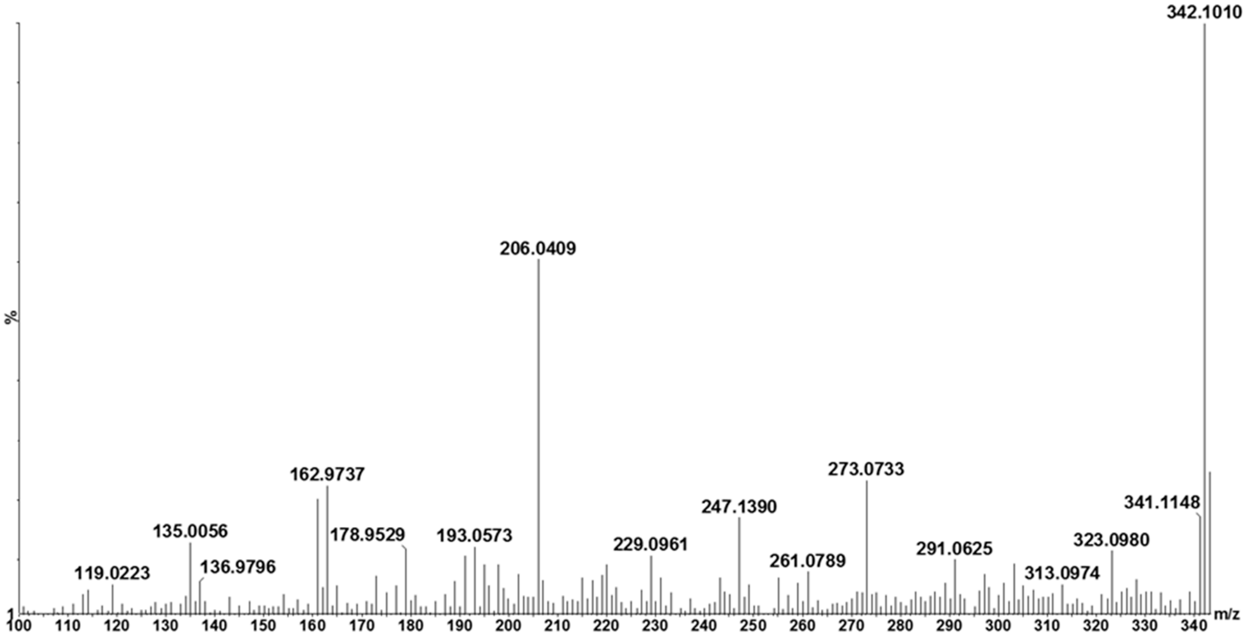

Figure S4. Mass spectra showing the fragmentation pattern for deoxyclovamide.
